# Factors Influencing Intentions of People with Hearing Impairments to Use Augmented Reality Glasses as Hearing Aids

**DOI:** 10.3390/bs14080728

**Published:** 2024-08-21

**Authors:** Liyuan Deng, Jiangjie Chen, Dongning Li

**Affiliations:** 1 School of Design, Jiangnan University, Wuxi 214122, China; 7210306013@stu.jiangnan.edu.cn; 2 College of Fine Arts, Huaqiao University, Quanzhou 362021, China; chenjiangjie@hqu.edu.cn

**Keywords:** hearing impairments, AR technology, AR hearing aid glasses, use intentions

## Abstract

The advent and progression of AR (augmented reality) technology, coupled with the emergence of AR hearing aid glasses, offer a novel opportunity for people with hearing impairments (PHI). This study aims to explore the intention of this population to employ AR hearing aid glasses as their choice of hearing aid device and the specific factors influencing their preference. This study utilized the partial least squares SEM (PLS-SEM) analytical method to create structural equation model for intentions of PHI to use AR glasses as hearing aids. Data were gathered from on-site experiences across multiple locations; a total of 189 valid questionnaires from individuals with varying degrees of hearing disabilities were used for statistical analysis. According to the data analysis results, we discovered that functionality quality, perceived interaction speed, and perceived usability significantly influence communication effectiveness. Further, communication effectiveness positively influences confidence and societal perception, and the latter has a positive impact on information. Both of these factors positively influence behavioral intention. Based on these findings, this study offers design recommendations for AR hearing aid glasses to cater to the specific needs of PHI, aiming to enhance their quality of life. Furthermore, this study provides pivotal insights for the prospective growth of this emerging industry.

## 1. Introduction

Hearing is one of the most important yet fragile functions in a person’s life, susceptible to various factors that can lead to hearing loss at any time for anyone [1,2,3]. As a result, hearing impairment has become the third most common cause of disability [4,5]. According to the World Hearing Report published by the World Health Organization, by 2021, over 5% of the global population suffered from hearing loss, and by 2050, it is projected that nearly 2.5 billion individuals will have different degree of hearing loss [6]. Hence, hearing impairment has gravely threatened global public health [7,8,9].

Unlike individuals who are deaf, people with hearing impairments (PHI), despite facing various difficulties due to hearing loss [10,11], can address these issues with the help of hearing aid products [12]. However, the adoption rate of assistive devices in the real-world context remains low among PHI [13,14]. Among them, hearing aids are the primary choice for hearing impairment rehabilitation [15,16,17], yet their adoption rate is less than 20% [18]. One of the main reasons for this phenomenon is the stigma associated with them [19,20]. Because hearing impairment is an invisible disability, the use of such assistive devices may inadvertently highlight it, leading to the bearer being labeled or perceived as disabled or an outsider in society [21,22,23,24,25,26]

Consequently, many individuals, before seeking medical intervention and receiving a diagnosis of hearing impairment, may prefer to experiment with devices that appear “normal” in any public setting, such as smart glasses [27,28,29], mobile phones [30,31], and smart earbuds like AirPods and Samsung Galaxy Buds Pro [32]. However, mobile phones as hearing-assistive agents are not suitable for everyday face-to-face communication, and smart earbuds, which merely amplify sounds, might risk further deterioration in hearing. In contrast, smart glasses offer advantages such as a natural appearance, sensory compensation, comfortable wear, and a broad range of applicability. They emerge as a superior choice. As dos Santos, Ferrari [33] pointed out, both glasses and hearing aids can assist those with hearing difficulties, but the stigma associated with glasses is significantly less.

With the advancement and proliferation of augmented reality (AR) technology, AR glasses demonstrate greater potential as hearing aid devices [28,33]. With AR technology, hearing-assistive glasses have transcended their basic function of merely integrating sound amplifiers into their frames. Instead, they now incorporate AR tech into the lens interface and visual effects, amalgamating features like real-time voice-to-text captions, specialized word prompts, spatial sound indicators, etc., into the AR glasses display system, enabling users with hearing impairments to communicate normally through AR glasses [29,34]. It is evident that AR hearing glasses have unique advantages in terms of technological functionality, social acceptance, and interactive experience. As a hearing aid device, they possess immense growth potential and a vast market outlook, holding significant social and economic value.

Given the anticipated significant increase in the number of PHI over the next 30 years, understanding their attitudes towards adopting and intention to use hearing-assistive technology is crucial [6,35]. However, current research on the adoption and usage of hearing aid devices mainly focuses on traditional hearing aids [36,37,38], and studies on the emerging product of AR hearing glasses are relatively scarce. Existing research on AR hearing aid glasses primarily centers on technological development, functional innovation, experience design, and product development [14,27,28,29,34,39], with insufficient exploration of whether PHI can truly accept and use AR hearing aid glasses. Most studies approach the issue from a single perspective, either technological functionality or sociocultural aspects [40,41,42,43,44], with few studies comprehensively exploring the willingness of PHI to adopt AR hearing aid glasses from an integrated perspective.

Therefore, this study, based on sociotechnical systems theory (STS), explores the factors and mechanisms influencing the intention of PHI to use AR hearing aid glasses from a comprehensive perspective encompassing technical, social, and individual dimensions. This research aims to provide insights and references for optimizing the design and enhancing the acceptance of AR hearing aid glasses, offering a new practical direction to improve the communication experience and social integration of PHI.

## 2. Theoretical Framework and Research Hypotheses

Sociotechnical systems theory (STS) is an interdisciplinary theory that provides a comprehensive perspective for understanding and designing technological systems by considering the complex interactions between technical, social, and individual factors [45,46]. The core idea of this theory is that various subsystems (technical, social, and individual) are interdependent and mutually influential. The design and implementation of any new technology, product, or service system must simultaneously consider the potential impacts on these subsystems to achieve optimal results [47,48]. This paper adopts the three-dimensional framework of sociotechnical systems theory—technical subsystem, social subsystem, and individual subsystem—as the theoretical foundation for the following reasons: On one hand, sociotechnical systems theory provides a comprehensive analytical framework for emerging issues related to hearing impairment behavior, hearing aid technology, and AR hearing glasses. On the other hand, research on hearing impairment and hearing aid technology is not merely a technical or individual health issue; it is a complex phenomenon that encompasses personal physiology, product technical functions, and social life [33,49]. Through the integrated perspective of sociotechnical systems theory, we can gain a more comprehensive understanding of the motivations and behavioral impact mechanisms of PHI using AR hearing glasses.

Based on the three dimensions of sociotechnical systems theory, this study explores the technical features of AR hearing aid glasses, the personal physiological characteristics of PHI, and the relevant factors of social life. It ultimately identifies seven factors, namely: functional quality, perceived interaction speed, perceived ease of use, communication effectiveness, social image, confidence, and behavioral intention. The operational definitions for each construct are presented in Table 1.

### 2.1. Function Quality

PHI use the functions of hearing aids to compensate for their hearing deficits [57]. The quality of these functions signifies the quality of sound information reception for them. Clear and consistent information is a significant influencing factor in interpersonal communication [58]. Therefore, the features of the hearing aids affect communication outcomes [59]. Many studies have also confirmed that the better the performance of hearing aids (such as noise suppression, clarity compensation, etc.), the better the communication outcomes for PHI in various scenarios [28,60,61,62,63]. Hence, this study proposes the following hypothesis:

**Hypothesis** **1** **(H1).**  *The functional quality of AR smart glasses has a positive impact on the communication outcomes of PHI*.

### 2.2. Perceived Interaction Speed

Research indicates that in interpersonal communication, the speed of interaction between people (speaking rate, cohesion speed, pause intervals, etc.) is one of the three main dimensions for achieving smooth communication. There is a significant relationship between the speed of interaction and the quality of communication [64,65,66]. For PHI, due to their hearing loss, they cannot communicate directly with those without hearing impairments but must do so mediated by hearing aids. Therefore, hearing aid products are considered an additional pair of ears for PHI [67]; the interaction speed of the hearing aids signifies the sensitivity and reaction speed of their “ears”. Gugenheimer, Plaumann [59] found that interruptions in conversation reduce communication quality. Conversely, if the device’s real-time interaction speed is fast enough, synchronous dialogue can be achieved [68], reducing interruptions during communication and thereby enhancing communication quality. Therefore, the faster the interaction speed of the AR smart glasses, the better the communication outcome. Based on this, this study proposes the following hypothesis:

**Hypothesis** **2** **(H2).**  *The perceived interaction speed of AR smart glasses by PHI has a positive impact on communication outcomes*.

### 2.3. Perceived Ease of Use

In this study, perceived ease of use refers to the degree of ease or difficulty that users with hearing impairments feel when using AR glasses. The literature has already confirmed that there is a significant relationship between perceived ease of use and communication [69,70]. Additionally, some studies have proven that the perceived ease of use of technology products designed for social communication has a positive impact on communication outcomes [71,72]. For example, Chan, Yong, and Harmizi [72] examined the relationship between the use of WhatsApp by students at private higher education institutions in Malaysia and interpersonal communication, confirming a positive correlation between perceived ease of use and interpersonal communication. Based on this, this study proposes the following hypothesis:

**Hypothesis** **3** **(H3).**  *The perceived ease of use of AR smart glasses has a positive impact on the communication of PHI*.

### 2.4. Communication Effectiveness

Being able to communicate with people who have unimpaired hearing is crucial for those with hearing impairments [59]. Many studies have confirmed that good communication interactions positively influence confidence enhancement [73,74]. Blood, Blood [75] pointed out in their study that stuttering adolescents may lack confidence in their communication abilities due to communication difficulties. Conversely, smooth communication can boost self-confidence. In the field of assistive hearing device research, enhancing the communication capabilities of users with hearing impairments through relevant products, technologies, and services can alleviate the anxiety and stress caused by fear [76,77], which in turn can further boost the confidence of users with hearing impairments [78,79,80].

There is a close relationship between communication and social image [81,82,83,84]. Lin and Bhattacherjee [54] confirmed in their study that users’ social communication with others in social networks positively impacts the creation of a favorable social image. Heffernan, Coulson [85] pointed out that the stigmatization and damage to the social image of PHI often result from communication difficulties. By achieving normal and free communication through technology without revealing their unique health status, PHI can avoid being seen as disabled, thus sidestepping stigmatized views and attitudes, and achieving equality and respect [86], which translates to a positive social image. Therefore, when AR hearing-assistive glasses help users with hearing impairments to communicate smoothly and avoid stigmatization, they can help these users establish a good social image. Hence, this study proposes the following hypotheses:

**Hypothesis** **4** **(H4).**  *Communication effectiveness has a positive impact on the shaping of the social image of PHI*.

**Hypothesis** **5** **(H5).**  *Communication effectiveness has a positive impact on the confidence of PHI*.

### 2.5. Social Image

Social image is defined as the extent to which users can obtain opinions and respect from their peers, family members, or others in their social relationships [87]. There is also a close relationship between social image and confidence. Confidence, in the context of personal motivation, explains why and to what extent people care about their self and social image, indicating that social image has a positive effect on confidence [88]. Lubis and Fazira [89] pointed out in their study that there is a significant positive correlation between social support (which includes the concept of social image) and confidence. In disability research, the role of social image in confidence is even more evident [90]. Simsek, Evli [91], in the context of chronic illness research, found a positive correlation between body image and confidence. Therefore, consistent with previous research, a good social image can enhance the confidence of the hearing-impaired group.

The study of social image in interactive products is of great significance. As the image of users comes from the opinions and views of others, users will thus decide on subsequent actions based on these views [92]. Some of the literature has confirmed that the social image produced by users using products or systems will significantly affect the subsequent intention to use [54,87,93]. Bispo and Branco [94] pointed out that the design of assistive technology for people with disabilities might change their social image. The same is true for hearing-assistive products—non-stigmatizing design may stimulate users’ desire and behavior to use [33]. AR smart glasses are generally not associated with one’s mental image of disability; this can reduce stigmatization, thus forming a good social image. To maintain this good social image, PHI may continue to choose to use AR glasses in the future. Based on the above discussion, this study proposes the following hypotheses:

**Hypothesis** **6** **(H6).**  *The social image formed by PHI during the use of AR glasses has a positive impact on their confidence*.

**Hypothesis** **7** **(H7).**  *The social image formed by PHI during the use of AR glasses has a positive impact on their behavioral intention*.

### 2.6. Confidence

Confidence can be defined as an individual’s belief in their abilities, a state of certainty about the success of a specific behavior [95]. The relationship between confidence and behavioral intention has been confirmed in many studies [96,97,98]. For instance, Kim, Ahn, and No [97] pointed out in their study that confidence in one’s physical health affects the choice and execution of behavior. Chai, Lin [98] found in their research that confidence in AI technology is positively correlated with the intention to continue using AI devices. In line with previous studies, this research believes that the confidence of PHI in the AR glasses technology assisting them in smooth communication has a positive impact on the intention to continue using AR glasses. Therefore, this article proposes the following hypothesis:

**Hypothesis** **8** **(H8).**  *The confidence gained from social communication through AR glasses has a positive impact on PHI’s intention to continue using AR glasses*.

**Figure 1:**
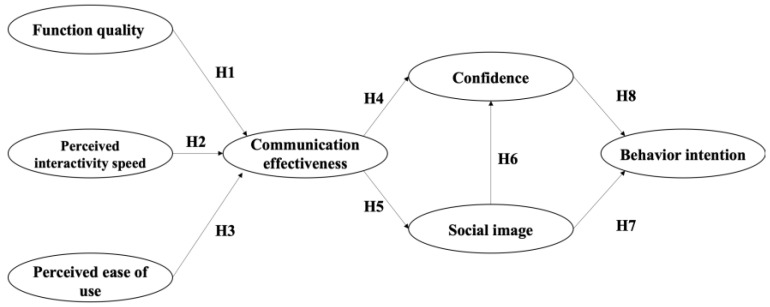
Research model.

Based on the discussions in the previous sections, this study formulates eight hypotheses. The path and hypothesis model are shown in Figure 1.

## 3. Questionnaire Design and Data Collection

### 3.1. Questionnaire Design

This study combined the product features of AR hearing aid glasses with the characteristics of the research to design a literature-validated questionnaire (see Table 2). After completing the initial questionnaire design, we conducted a pretest with 30 participants and adjusted the questionnaire based on their feedback. The final version included basic information and an assessment of factors influencing the use of AR hearing aid glasses. (using a Likert’s seven-point scale).

### 3.2. Data Collection

The research and experimentation for this study spanned eight months. During the preliminary preparation phase from January to April 2023, we conducted an initial screening of hearing-impaired subjects, visited multiple locations, tested and selected experimental tools, and designed and evaluated the questionnaire. From May to August 2023, we carried out formal questionnaire surveys at hearing centers and service institutions in Wuxi, Xiamen, Hangzhou, and other cities in China, using both offline experiences and online questionnaires. We employed a cluster sampling technique. Conducting surveys through established hearing centers and service institutions provided practical benefits, as it allowed for us to efficiently recruit a large number of participants within the given timeframe. Additionally, this approach enabled us to capture a broad range of experiences and perspectives from participants across various locations.

We used the LEION HEY AR smart glasses as the testing tool and invited participants to complete the questionnaire after experiencing the product for more than 15 min. After screening, a total of 189 valid questionnaires were collected. The demographic information of the respondents is shown in Table 3.

## 4. Data Analysis and Results

This study employs the partial least squares structural equation modeling (PLS-SEM) research method. As a flexible multivariate analysis approach, PLS-SEM is utilized for constructing and validating complex structural equation models [107]. Therefore, this study utilized the PLS-SEM algorithm in SmartPLS 4 V4.0.9.2, employing a weighted path scheme, with a maximum of 3000 iterations and default initial weights. Further, we used path analysis techniques to verify the eight proposed hypotheses. Finally, we integrated the empirical data and conducted an in-depth analysis within the existing theoretical framework, and then drew rigorous conclusions.

This study employed the SmartPLS software to calculate the reliability and validity of constructs, specifically considering three indicators: Cronbach’s alpha, composite reliability, and average variance extracted (AVE). As shown in Table 4, the Cronbach’s alpha value for each construct in this study exceeded 0.7, and the rho_c value also exceeded 0.7, indicating that all the constructs used have good reliability. Next, this study used AVE to measure convergent validity. The usual standard is for AVE to be greater than 0.5. All the indicators in this study meet this requirement [108]. Therefore, the constructs in this study exhibit good convergent validity.

If the square root of the AVE value of each construct is greater than the correlation coefficients between itself and other constructs, it suggests that the scale has good discriminant validity [109]. As shown in Table 5, the bold numbers on the diagonal represent the square root of the AVE values for each construct, which are all greater than the correlation coefficients, which indicates that there is good discriminant validity between the constructs used in this study.

This study also uses the HTMT (heterotrait/monotrait ratio) method to evaluate the discriminant validity of the constructs [110]. According to previous research, the HTMT value must be less than 0.9 [111]. As shown in Table 6, the HTMT values for all constructs are less than 0.9. Therefore, all constructs in this study have good discriminant validity.

Also, during this stage, additional measures were evaluated, namely, the determination coefficient (R^2^) and predictive relevance (Q^2)^ [108]. As shown in Table 7, the R^2^ values between the constructs range from 46.9% to 66.9%. R^2^ values greater than 26% are considered significant [112]. In this study, the Q^2^ values of the constructs (calculated only for dependent variables) are all non-zero. Therefore, the hypothesized model in this study has acceptable predictive relevance [113]. Additionally, indicators such as the standardized root mean square residual (SRMR) and the normed fit index (NFI) were used to evaluate the fit of the structural equation model (see Table 8). The SRMR value is 0.082, which is below the threshold of 0.10, and the NFI value is 0.804, where an NFI value close to 1 indicates a good model fit. These fit indices suggest that the hypothesized model proposed in this study performs well in explaining the data structure.

**Figure 2:**
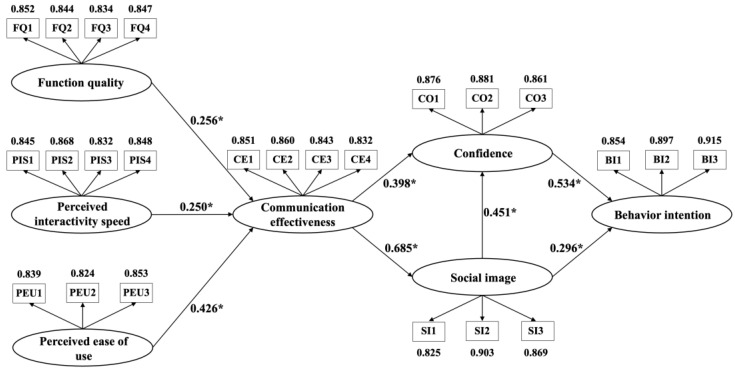
Results of path analysis. (* The level of significance is below 0.05).

The bootstrapping procedure (with 5000 bootstrap resampling) was applied to further test the proposed hypotheses. The results of the model path analysis are shown in Figure 2, and the regression coefficient results of the structural equation model are shown in Table 9. The results indicate that all hypotheses of this study are supported. Furthermore, the full collinearity test results show that the variance inflation factor (VIF) ranges between 1.000 and 2.674. All the VIFs are below the recommended threshold of 5 [108], confirming that there is no multicollinearity issue in the estimated model of this study.

## 5. Discussion

According to the research results, function quality and perceived interactivity speed have a significant positive impact on communication effectiveness (H1 and H2 are supported). The previous literature has pointed out that hearing aids can be considered an additional pair of ears for users with hearing impairments [67]. The quality and speed of their functions are equivalent to the clarity and sensitivity of these “ears”. AR hearing aid glasses serve as an intermediary for PHI to interact with external sounds. The higher the functional quality of AR hearing aid glasses, the clearer the external sounds they read, and the more accurate the converted text information, leading to better communication effectiveness for the user. Similarly, if AR hearing aid glasses can quickly capture, process, and present visual information, enabling PHI to swiftly acquire conversation content, speech-to-text information, and respond accordingly, this instant feedback helps reduce delays and misunderstandings in communication. This allows for a synchronized rhythm with others, avoiding interruptions in conversations, making the entire communication process more natural, relaxed, and smooth [59], thereby enhancing the communication effectiveness for PHI.

Perceived ease of use has a significant positive impact on communication effectiveness (H3 is supported). Unlike traditional hearing aids, which users cannot control independently, AR hearing aid glasses offer more functions, meaning there may be many aspects requiring user operation. Although most functions are set up before communication interactions, the complexity of social situations and the unpredictability of human behavior can lead to changes in social communication conditions and needs [114]. This, in turn, necessitates adjustments to the AR hearing aid glasses, such as font size, color, and contrast, as well as turning on the device or adjusting the volume. If these adjustments can be easily made during conversations, PHI can focus more on the communication itself, thereby enhancing the effectiveness and experience of their interactions. 

It is worth noting that even though function quality, perceived interaction speed, and perceived ease of use all have significant positive impacts on communication effectiveness, and perceived ease of use has a stronger influence on communication effectiveness (0.426 > 0.256 > 0.250). The reason for this might be related to the unique ways PHI receive information and their cognitive processing methods. PHI are not completely deaf; they still retain some degree of hearing ability. Before using hearing aids, PHI might intentionally or unintentionally employ various skills to complement this portion of sound information. For example, they imagine and reason to fill in the details not fully heard in conversations, especially for unclear words and phrases. They may also use non-verbal strategies to enhance communication, such as observing the speaker’s lip movements, body language, facial expressions, and contextual environment to infer the meaning, emotions, and attitudes in the conversation [115,116,117]. By utilizing these techniques, PHI can combine their existing knowledge, memory, and experience to partially fill in the missing information and deduce the logical conclusions of the conversation [118]. Thus, for AR hearing-assistive glasses, even if the accuracy of voice-to-text isn’t very high or the conversion speed is slow, individuals can still construct a complete dialogue scene using their imagination and reasoning skills. In contrast, the perceived ease of use of AR hearing-assistive glasses has a more significant effect on communication. On the one hand, the usage method of AR hearing aid glasses differs significantly from traditional hearing aids. Traditional hearing aids require little to no manual operation during daily use, whereas AR hearing aid glasses necessitate user interaction. This interaction can occur at any stage before, during, or after communication, emphasizing the importance of ease of use. On the other hand, the user’s operation of the glasses is explicit and visible to both parties. If the glasses are challenging to use, users might need to adjust them repeatedly during conversations, not only wasting time and causing communication delays but also diverting their attention. Not focusing on the interlocutor during a conversation is considered impolite and might make the other party feel disrespected [119]. Therefore, the influence of perceived ease of use on communication effectiveness can create a chain reaction, much like the butterfly effect, resulting in a stronger impact. 

Secondly, communication effectiveness has a significant positive impact on both social image and confidence (H4 and H5 are supported). With the assistance of AR glasses, effective two-way communication enables clear understanding and expression of information, preventing many awkward situations such as being unable to participate in group conversations, missing important information, or not responding promptly. This helps to avoid feelings of marginalization and inferiority, thereby enhancing conversational confidence. Furthermore, the stigmatization and negative social image of those with hearing impairments are often directly related to communication challenges [85]. With the support of AR hearing-assistive glasses, PHI can establish effective two-way communication, build a positive image, and gain more recognition, respect, and equality in social life, education, and career development [86]. 

Social image has a significant positive impact on confidence (H6 is supported), which is consistent with the findings of previous studies [89,90,91]. According to Tajfel and Turner [120], when individuals feel recognized and affirmed within their social group, their confidence is boosted. PHI often do not consider themselves having a disability [121]. Therefore, they desire to establish an image of capability equal to those with without impaired hearing. However, this desire often contradicts the use of hearing-assistive devices because the distinctive appearance of most of these devices inadvertently reveals their hearing deficiencies. Unlike other hearing aids that might expose one’s hearing challenges, AR glasses possess high social acceptability and low stigmatization. They can enhance communication abilities without disclosing hearing impairments. Once this positive social image is recognized by others, it can boost positive emotions and confidence.

Lastly, both social image and confidence have a significant positive impact on the intention to use (H7 and H8 are supported). In social life, individuals not only perceive themselves as independent entities but also as members of social groups, and they seek recognition from these groups [120]. Therefore, the social image is crucial. When PHI gain a wholesome social image by using AR glasses, integrating them into the mainstream group, they are more likely to accept and adopt hearing-assistive technology. Conversely, devices that expose their hearing deficiencies are likely to be rejected [122]. Furthermore, compared to social image, confidence has a stronger impact on the intention to use (0.534 > 0.296). This might be due to confidence serving as an intrinsic factor, exerting a more direct inner drive on usage intention, while the social image, as an external factor, primarily plays a supporting role. The construction of social image is rooted in others’ perceptions and attitudes, an external push. This external force typically undergoes self-awareness, filtration, absorption, and rational analysis before it internalizes and influences behavior. Throughout this process, users filter some external factors based on their self-perception, reducing the impact of these factors to some extent. In contrast, confidence, being an intrinsic driver, can directly influence behavior choices [123], and hence has a more potent influence. 

## 6. Contributions and Limitations

This study, based on sociotechnical systems theory, explores the factors influencing the intention of PHI to use AR glasses as hearing aid devices from a comprehensive perspective of technological, social, and individual levels. It identifies the real needs and decision-making mechanisms of PHI, enriching the theoretical foundation of the hearing aid design field and providing practical guidance and insights.

### 6.1. Theoretical Contributions

(1). This study enriches the field of behavioral research in hearing impairment and design. By introducing sociotechnical systems theory into the study of hearing impairment behavior, it explores how the interaction between technological, social, and individual dimensions affects the experience of PHI using AR hearing aid glasses, thereby influencing their subsequent adoption intentions. This research provides a new explanatory pathway for the behavior of PHI using AR hearing glasses and offers a theoretical framework for further understanding and recognizing the deep needs of PHI. It also provides theoretical support for the development and design of hearing impairment treatments, services, and related products. Furthermore, this study helps overcome the limitations of technocentrism and partial perspectives in hearing impairment research.
(2). Through quantitative analysis, this study elucidates the intrinsic mechanisms and decision-making pathways for PHI choosing AR glasses as hearing aid devices. Specifically, it identifies that social image and confidence are the decisive factors influencing users’ intention to use AR hearing aid glasses, while the technical aspects of functional quality, perceived interactivity speed, and perceived ease of use are merely necessary prerequisites and foundations and do not directly determine the intention to use AR hearing aid glasses. This finding aligns with the general direction of existing research on hearing impairment and hearing aid technology, providing further empirical evidence. Additionally, by examining specific aspects and more tangible product targets, this study clarifies the intrinsic relationships and influencing mechanisms between variables at the technical, social, and individual levels and the intention to use AR hearing glasses. This provides more targeted theoretical guidance for strategy formulation and practical implementation in the field of hearing aid technology and services.

### 6.2. Practical Contributions

In terms of practical implications, the results of this study make tangible contributions to expanding the market for AR hearing-assistive glasses and sustainable development. By clarifying various factors influencing the use of AR glasses as hearing-assistive devices by PHI, this study provides a research foundation and guiding direction for the development of AR hearing-assistive glasses and other hearing-assistive products. It offers recommendations and design strategies for optimizing their design, mainly including the following:

(1). In the development of AR hearing-assistive glasses, special attention should be given to the interactive ease-of-use design of the AR glasses. The basic operations of the AR glasses should be simplified to prevent users from abandoning their use due to difficulty. Consideration can even be given to employing the most advanced intelligent proactive interactive methods, allowing for the device to autonomously complete interactions based on circumstances. This avoids tedious operations during communication and allows for users to focus on communicating, thereby enhancing the communication experience.
(2). It is essential to integrate as many functions as possible, such as noise reduction, multi-directional microphone pickups, and adaptive light brightness adjustments and ensure the high quality of these features to assist PHI in communicating normally in various environments. It is also vital to improve the interaction speed of AR hearing-assistive glasses, such as the response speed for keyword activation and the speed of voice-to-text, so that PHI can maintain a consistent rhythm with their counterparts during communication.
(3). The design of AR glasses should closely resemble regular glasses used in daily life. The lens can adopt a single-sided design to ensure the virtual interface is not visible to others, thus avoiding excessive attention. This helps PHI rid themselves of the “special” label, thereby building social confidence. It is also essential to design the text information’s display size, color, transparency, and position well, so users can clearly view the text while not obscuring the eyes and non-verbal actions of others. This facilitates more eye contact during communication, allowing for users to express their sincerity, earn the respect of others, establish a positive social image, and better integrate into various aspects of life, education, and work.

### 6.3. Research Limitations

While this study offers valuable contributions both theoretically and practically, there are still some limitations. On one hand, due to objective constraints such as culture, funding, geography, and time, we encountered many obstacles when trying to locate PHI, making it difficult to expand our sample size. On the other hand, since AR hearing-assistive glasses are an emerging product, most of our respondents were completely unfamiliar with them. This may lead to self-report biases and potential errors in data validity. Although we made efforts to minimize these biases by providing product descriptions, allowing participants to try the glasses and offering guidance as necessary before they filled out the questionnaire, we still recognize that this limitation could mislead our study’s results. Therefore, when interpreting and promoting the findings of this study, potential uncertainties arising from data biases should be carefully considered. Moreover, there are numerous areas for further detailed research. For instance, PHI from different age groups may have varied attitudes towards AR glasses. Our study, with its limited sample, did not differentiate the intention to use based on age groups. Secondly, different genders might have different preferences for the design and aesthetics of hearing-assistive devices. Thirdly, acceptance of AR hearing-assistive glasses might be influenced to different degrees in various countries and cultural environments. Future research can build upon the foundation of this study and further segment personal attributes and societal scopes, allowing for more targeted and in-depth exploration of AR hearing-assistive glasses.

## Figures and Tables

**Table 1:** Operational definitions of constructs.

Construct	Operational Definition	Source
Function quality	The completeness and quality of functions of AR smart glasses.	Wang and Chuan-Chuan Lin [50]
Perceived interaction speed	The response speed of AR smart glasses as perceived by PHI.	Steuer, Biocca and Levy [51]
Perceived ease of use	The ease of use of AR smart glasses as perceived by PHI.	Davis [52]
Communication effectiveness	The effectiveness and quality of interactions and communication between PHI and others.	Sweeney, Morrison [53]
Social image	The views and respect that PHI receive from others.	Lin and Bhattacherjee [54]
Confidence	The confidence of PHI that AR glasses can assist them in communication.	Wilkes [55]
Behavioral intention	The intention of PHI to use AR smart glasses as hearing aids.	Taylor and Todd [56]

**Table 2:** Measurement scale of influencing factors of PHI using AR hearing assistant glasses.

Constructs	Items	Content	Source
Function quality	FQ1	I feel that the speech-to-text accuracy of AR glasses is very high.	Shanahan, Tran and Taylor [99], Wang, Wang [100]
	FQ2	I feel that the speech-to-text quality of AR glasses is high.	
	FQ3	I believe that the information output from the AR glasses is reliable.	
	FQ4	I believe the functionality quality of the AR glasses meets my requirements.	
Perceived interaction speed	PIS1	I feel that the speech-to-text speed of AR glasses is very fast.	Huang, Chiu [101]
	PIS2	I believe the process of speech-to-text with AR glasses is very smooth.	
	PIS3	I believe the speech-to-text speed of AR glasses is almost synchronous with the speed of the other person’s speech.	
	PIS4	I feel that the response speed of the AR glasses is fast when communicating with others.	
Perceived ease of use	PEU1	Learning how to use AR glasses is easy for me.	Khlaisang, Songkram [102]
	PEU2	Interacting with AR glasses doesn’t require much effort.	
	PEU3	I can use AR glasses proficiently in communication.	
Communication effectiveness	CE1	With the help of AR glasses, I communicate with others more easily and comfortably.	Sweeney, Morrison [53], Lam and Campbell [103]
	CE2	With the assistance of AR glasses, I can communicate better with others.	
	CE3	AR glasses help me understand others more effortlessly.	
	CE4	With the assistance of AR glasses, I can communicate with others smoothly and without barriers.	
Social image	SI1	By using AR glasses, I have shed the image of being hearing-impaired.	Yang, Yu [104]
	SI2	By using AR glasses, others see me as a normal person.	
	SI3	Using AR glasses gives others the impression that I am a regular individual.	
Confidence	CO1	With the help of AR glasses, I now have the confidence to handle the entire communication process.	Hong, Hwang [96]
	CO2	Compared to before, with the help of AR glasses, I believe my communication performance is better.	
	CO3	Compared to before, with the assistance of AR glasses, I will communicate with others more confidently.	
Behavioral intention	BI1	I am willing to use AR glasses as my hearing assistance device.	Rajeh, Abduljabbar [105], Hong, Lin and Hsieh [106]
	BI2	I would recommend AR glasses to my hearing-impaired friends.	
	BI3	I will continue to use AR glasses in the future.	

**Table 3:** Demographic information of the respondents.

Category	Group	Number	Percentage
Gender	Male	73	38.624%
	Female	116	61.376%
Age	<18	12	6.349%
	18–30	100	52.910%
	31–40	36	19.048%
	41–50	24	12.698%
	51–60	14	7.407
	>60	3	1.587
Education	High school or technical secondary school and below	55	29.101%
	junior college	63	33.333%
	Undergraduate	65	34.392%
	Graduate and above	6	3.175%
Degrees of hearing impairment	Mild	107	56.614%
	Moderate	77	40.741%
	Severe	5	2.646%

**Table 4:** Loadings, AVE, composite reliability, and Cronbach’s alpha.

Constructs	Items	Loadings	Cronbach’s Alpha	Composite Reliability (rho_a)	Composite Reliability (rho_c)	AVE
Function quality	FQ1	0.852	0.866	0.869	0.909	0.713
	FQ2	0.844				
	FQ3	0.834				
	FQ4	0.847				
Perceived interaction speed	PIS1	0.845	0.87	0.874	0.911	0.72
	PIS2	0.868				
	PIS3	0.832				
	PIS4	0.848				
Perceived ease of use	PEU1 PEU2 PEU3	0.839	0.79	0.797	0.877	0.703
		0.824				
		0.853				
Communication effectiveness	CE1	0.851	0.868	0.868	0.91	0.717
	CE2	0.86				
	CE3	0.843				
	CE4	0.832				
Social image	SI1	0.825	0.833	0.838	0.9	0.75
	SI2	0.903				
	SI3	0.869				
Confidence	CO1	0.876	0.843	0.845	0.905	0.761
	CO2	0.881				
	CO3	0.861				
Behavioral intention	BI1	0.854	0.867	0.869	0.919	0.791
	BI2	0.897				
	BI3	0.915				

**Table 5:** Discriminant validity: Fornell–Larcker criterion.

	FQ	PIS	PEU	CE	SI	CO	BI
**FQ**	**0.844**						
**PIS**	0.771	**0.848**					
**PEU**	0.609	0.606	**0.839**				
**CE**	0.708	0.705	0.733	**0.847**			
**SI**	0.593	0.593	0.571	0.685	**0.866**		
**CO**	0.589	0.569	0.616	0.707	0.724	**0.873**	
**BI**	0.589	0.586	0.645	0.763	0.683	0.748	**0.889**

Note: Bold numbers is the square root of the AVE values for each construct.

**Table 6:** Discriminant validity: heterotrait/monotrait ratio (HTMT).

	FQ	PIS	PEU	CE	SI	CO	BI
**FQ**							
**PIS**	0.887						
**PEU**	0.731	0.721					
**CE**	0.812	0.808	0.878				
**SI**	0.696	0.695	0.698	0.805			
**CO**	0.684	0.660	0.755	0.825	0.861		
**BI**	0.675	0.672	0.772	0.879	0.801	0.875	

**Table 7:** Results of R^2^ and Q^2^.

Construct	R^2^	Q^2^
Communication effectiveness	0.669	0.472
Social image	0.469	0.348
Confidence	0.608	0.454
Behavior intention	0.602	0.471

**Table 8:** Model fit indices.

Indices	Estimated Model
SRMR	0.082
d_ULS	2.007
d_G	0.598
Chi square	648.539
NFI	0.804

**Table 9:** Path coefficients and the results of the significance tests.

Hypothesis	Path	Std Beta	*t* Statistics	*p* Values	VIF	Results
H1	FQ → CE	0.256	3.329	0.0001	2.674	Support
H2	PIS → CE	0.250	3.449	0.0001	2.655	Support
H3	PEU → CE	0.426	7.618	0.0000	1.715	Support
H4	CE → CO	0.398	6.298	0.0000	1.882	Support
H5	CE → SI	0.685	16.194	0.0000	1.000	Support
H6	SI → CO	0.451	7.337	0.0000	1.882	Support
H7	SI → BI	0.296	3.683	0.0000	2.101	Support
H8	CO → BI	0.534	7.314	0.0000	2.101	Support

## Data Availability

The data that support the findings of this study are available from the corresponding author upon reasonable request.
